# Principles of Epilepsy Management for Women in Their Reproductive Years

**DOI:** 10.3389/fneur.2020.00322

**Published:** 2020-04-28

**Authors:** Rebecca Spiegel, Heidy Merius

**Affiliations:** Department of Neurology, Stony Brook Medicine, Stony Brook, NY, United States

**Keywords:** epilepsy, seizures, antiseizure medications (ASMs), women with epilepsy (WWE), reproductive years, teratogenic effects AEDs, major congenital malformations (MCM), neurocongnitive development

## Abstract

In the United States, there are over one million women with epilepsy (WWE) in their childbearing years. Pregnancy can be challenging for this population. A number of international registries have documented that children born to these women are at increased risk for major congenital malformations (MCM), lower intelligence quotient scores and neurodevelopmental disorders, when the mother is managed on antiseizure medications (ASMs). To prevent poor neonatal outcomes for this population, safe and thoughtful management strategies are necessary. We propose to divide these management strategies into five principles. These include (I) choosing suitable ASMs for the patient's seizure type, (II) choosing an ASM with the least teratogenic and cognitive side effects, (III) dosing at the lowest possible effective dosage, (IV) selecting the best ASM regimen as promptly as possible, even before a woman has her first menses, and (V) supplementing these patients with folic acid in order to try to enhance cognition and reduce neural tube defects.

## Introduction

In the United States, there are over one million WWE in their childbearing years (1). Because of the reproductive potential of these women their management can often differ from males and post-menopausal women.

Management of seizures is traditionally guided by the classification of seizures as focal or generalized in onset. Thankfully, there are ASMs that can treat seizures in each classification. That selection is then narrowed down further in WWE based on the teratogenicity potential of these ASMs that is available from the various pregnancy registries. These registries include the North American Pregnancy Registry, The UK & Ireland Epilepsy and Pregnancy Register, EURAP Registry (includes 44 countries all around the world) and the Australian Registry.

Along with an increase of MCM some ASMs can also lead to lower intelligence quotient scores, and neurodevelopmental disorders (1). Unintended pregnancies further complicate this risk as they often lead to inadequate or delayed initiation of prenatal care and an increased risk for fetal exposure teratogenic substances such as alcohol and nicotine (2). In 2011, there were 45 unintended pregnancies for every 1,000 women aged 15–44 years (3). Similar rates are reflected worldwide in other developed countries, but are substantially higher in developing countries at 65 unintended pregnancies for every 1,000 women age 15–44 years (4). It is thus evident that WWE in their reproductive years require different management strategies to improve their healthcare outcomes as well as the health of their potential offspring.

## The Five Principles

### Principle I: Choosing the Best ASMs for the Patient's Seizure Type

Clarifying the type of seizure each patient experiences guides the practitioner in selecting the appropriate ASMs. ASMs are generally categorized as broad spectrum and narrow spectrum. Broad spectrum ASMs are defined as agents that can be effective for both focal and generalized onset seizure types. Narrow spectrum ASMs, on the other hand, are traditionally only used in patients whose seizures arise from a specific focus or foci.

Broad spectrum ASMs include valproic acid (VPA), lamotrigine, topiramate and levetiracetam. Some ASMs such as clobazam and rufinamide are FDA-approved for only certain types of generalized seizures but are frequently used “off label” as broad spectrum agents for all generalized seizure and focal seizure types. In addition, other ASMs such as brivaracetam, felbamate, zonisamide, and lacosamide are FDA approved to only treat focal seizures but are often used off label as broad spectrum ASMs for all generalized seizure types.

There are, however, narrow spectrum ASMs that can in fact worsen certain types of generalized seizures and are thus used to treat mostly focal seizures. These ASMs include carbamazepine, oxcarbazepine, phenytoin, pregabalin, and gabapentin. The focal seizures these ASMs treat can range from focal aware seizures, focal seizures with impaired awareness as well as focal to bilateral tonic clonic seizures (5).

Once the type of seizure is identified the practitioner can then narrow down the ASM list to the ones most suitable for the patient's seizure type.

### Principle II: Choosing an ASM or ASMs With the Least Teratogenic and Cognitive Side Effects

We now have a variety of ASMs we can prescribe regardless of the seizure type the patient has. For WWE in the reproductive age group, the practitioner needs to further narrow the list of ASMs that are most appropriate in this patient population based on the ones that have the lowest rates of MCM. MCM are structural abnormalities that usually require surgical, medical, and cosmetic services (i.e., cleft lip, cleft palate, malformed limbs, neural tube defects, and cardiac abnormalities).

Since the 1990s birth outcomes of children born to WWE have been closely monitored through different pregnancy registries. Despite differences in methodology, the registries have generally reported similar findings and have all noted that exposure to VPA poses the greatest risk for MCMs. They have also shown that both lamotrigine and levetiracetam have a relatively low potential for MCMs. These findings have led to a marked difference in the way we now prescribe ASMs to WWE in the reproductive age group, with lamotrigine and levetiracetam being the most prescribed ASMs in many countries across the world (6, 7).

#### Monotherapy vs. Polytherapy

Polytherapy has been shown to increase the risk for major congenital malformation, however recent studies are proving this depends upon specific ASM combinations. The UK & Ireland Epilepsy and Pregnancy Register revealed highest MCM rates when levetiracetam was combined with VPA or carbamazepine (6.90%; 95% CI 1.91–21.96% and 9.38%; 95% CI 4.37–18.89%, respectively) and the lowest risk when combined with lamotrigine (1.77%; 95% CI 0.49–6.22%) (8). Similarly, EURAP data revealed highest MCM rates with ASM combinations that included VPA (9.1%; 95% CI 3.4–19.0% with lamotrigine and 15.4%; 95% CI 6.5–29.3% with carbamazepine), but not when carbamazepine was combined with all other non-VPAASMs (2.5%; 95% CI 1.1–4.36%) (9). Ultimately, avoiding polytherapy especially in combinations that include VPA is strongly recommended when possible.

#### Neurocognitive Considerations

While pregnancy registries focus on MCM, there has been growing evidence for the adverse effects of ASMs on neurocognitive development. Poorer cognitive ability has been proven with *in utero* exposure to specific ASMs. Children exposed to ASMs (monotherapy lacosamide, carbamazepine, lamotrigine, other, and polytherapy) had statistically poorer scores for overall development in comparison to children not exposed to ASMs (*p* < 0.001) (10). Differences in overall developmental ability were observed in children exposed to monotherapy VPA *in utero* when compared to the control group (*p* < 0.001). In addition, *in utero* exposure to VPA showed statistically more children below average range (score <84) for overall early development in comparison to control group (8%, *p* < 0.001). Similar results on neurocognitive development have been found in other studies where VPA and lamotrigine led to a statistically significant increased risk of having abnormal emotional and behavioral development (11). Conversely, carbamazepine was not associated with increased risk of emotional or behavioral development. Other neurodevelopmental finding showed increased risk of autism spectrum disorders and significantly reduced IQ scores with VPA in comparison to other ASMs (12–14).

### Principle III: Dosing to Reduce Complications

Pregnancy registries' outcomes have not only guided us about which ASMs are considered the safest to prescribe for WWE in their reproductive years but have also shed light on ASM dosing in this population.

Dose-dependent risks were observed in the UK & Ireland Epilepsy and Pregnancy Register and the EURAP Registry with a higher risk of MCM at the higher ASM dosages (15). This is particularly true for women taking an ASM such as VPA (>1,000 mg/day in the first trimester, Table 1) (16, 18, 22). Higher rates of MCM were observed between low dose and high dose VPA and low dose and high dose carbamazepine, but not markedly different for low and high doses of lamotrigine (Table 1) (16). More recently, a Cochrane systematic review also supported dose-dependent major malformation risk for carbamazepine (>700 mg/d), lamotrigine (>325 mg/d), phenobarbital (>80 mg/d), and VPA>650 mg/d) (23, 24). Higher doses of VPA (preconception dose of >900 mg) were also associated with poorer overall developmental scores (*p* < 0.001) (10).

**Table 1:** Major congeniital malformation rates from the UK & Ireland Epilepsy and Pregnancy Register, EURAP, Australian Pregnancy Register, and North American Antiepileptic Drug Pregnancy Registry.

**Registry**	**MCM rate following antiepileptic drug exposure**				
	**Valproate**	**Carbamazepine**	**Lamotrigine**	**Levetiracetam**	**Topiramate**
UK & Ireland Epilepsy and Pregnancy Register (8, 16, 17)	Dose: 0– ≤ 600 mg 24/476 5.0% CI (3.4–7.4%)	Dose: 0– ≤ 500 mg 14/721 1.9% CI (1.2–3.2%)	Dose: 0– ≤ 200 mg 24/1,143 2.1% CI (1.4–3.1%)	2/304 0.7% CI (0.2–2.5%)	3/70 04.8% CI (1.7–13.3%)
	Dose: >600– ≤ 1,000 mg 26/426 6.1% CI (4.2–8.8%)	Dose: >500– ≤ 1,000 mg 20/739 2.7% CI (1.8–4.1%)	Dose: >200– ≤ 400 mg 16/665 2.4% CI (1.5–4.0%)		
	Dose: >1,000 mg 31/297 10.4% CI (7.4–14.4%)	Dose: >1,000 mg 9/170 5.3% CI (2.7–9.5%)	Dose: >400 mg 9/276 3.4% CI (1.9–6.5%)		
EURAP (7, 18)	Dose: ≤650 mg/day 38/600 6.3% CI (4.5–8.6%)	Dose: ≤700 mg/day 58/1,276 4.5% CI (3.5–5.8)	Dose: ≤35 mg/day 46/1,870 2.5% CI (1.8–3.3%)	Dose: 250–4,000 mg/day 17/599 2.8% CI (1.7–4.5%)	Dose: 25–500 mg/day 6/152 3.9% CI (1.5–8.4%)
	Dose: >650– ≤ 1,450 mg/day 75/666 11.3% CI (9.0–13.9%)	Dose: >700 mg/day 49/681 7.2% CI (5.4–9.4%)	Dose: >325 mg/day 28/644 4.3% CI (2.9–6.2%)		
	Dose: >1,450 mg/day 29/115 25.2% CI (17.6–34.2%)				
Australian Pregnancy Register (19)	43/290 14.8% CI (2.11–12.95%)	24/409 5.9% CI (0.8–5.33%)	20/406 4.9% CI (0.66–4.55%)	5/139 3.6% CI (0.37–4.29%)	1/53 1.9% CI (0.09–5.96%)
North American Antiepileptic Drug Pregnancy Registry (20)	30/323 9.3% CI (6.4–13.0%)	31/1,033 3.0% CI (2.1–4.2%)	31/1,562 2.0% CI (1.4–2.8%)	11/450 2.4% CI (1.2–4.3%)	15/359 4.2% CI (2.4–6.8%)

*Table adaptation obtained from Elsevier, Kinney and Craig (21)*.

*CI, 95% Confidence interval*.

### Principle IV: Promptly Selecting the Best ASM Regimen

The rate of unintended pregnancies is not only high in the general population but also in WWE. Thus, promptly selecting the best ASM regimen (based on the above principles) when a woman is nearing the reproductive years is very important. Herzog et al. found that of the 437 women who reported getting pregnant after seizure onset, 78.9% of them reported having at least one unintended pregnancy (25). Sadly, by the time a woman misses her first period after conception, primary neural tube formation (which occurs in the first 4 weeks of gestation) has already taken place and potential neural tube damage may be irreversible.

Additionally, changing medications while the patient is pregnant exposes the patient and her fetus to the unknown effectiveness of the new ASM, thereby, placing the woman at risk of having seizures during pregnancy. Epileptic seizures were found to be associated with a 1.36-fold increased risk for low birth weight infants, 1.63-fold increased risk for preterm delivery, and 1.37-fold increased risk for small-for-gestational-age infants in a nationwide population-based study for 1,016 Taiwanese women with epilepsy (26). Moreover, the effects of generalized tonic-clonic seizures during pregnancy are particularly worrisome as they can lead to fetal asphyxia, fetal bradycardia, reduced uterine contractions, direct injury (both to the mother and fetus), and fetal demise.

### Principle V: Supplement All WWE in the Reproductive Age Group With Folic Acid

Folic Acid exposure has been shown to prevent neural tube defects in the general population (27, 28). Given that ASMs such as VPA can interfere with neural tube development it has become standard of care among epileptologists, to provide relatively high dosing of folic acid in the range of 2–5 mg to mitigate those effects. Despite this common practice, it is important to note that it has not been proven, thus far, that folic acid prevents neural tube defects in women taking ASMs (28–30). It is possible that the neural tube deficits that are linked to ASMs are due to mechanisms that do not involve folic acid metabolism (28, 29, 31).

Recent literature, however, has shown that folic acid may be beneficial in reducing the risk of autistic traits, enhancing children's IQ, and language development if the mother has taken folic acid for 4 weeks pre-gestation and post-conception (32–34). What is not clear, is the exact dosage of folic acid that is needed to improve cognitive outcome. Best cognitive outcomes were observed in children of women taking at least 0.4 mg/day of folic acid in the NEAD study and at least 1 mg/day in the Norwegian Mother and Child Cohort Study (32, 33). Since the data set is rather limited, we still support the use of about 4 mg of folic acid in patients who are taking ASMs that impair folic acid absorption (such as phenytoin, carbamazepine, and phenobarbital, as these can cause a deficiency of folic acid by interfering with the way it is absorbed). Patients taking VPA or who have a history of neural tube defects in their family should also be supplemented with about 4 mg of folic acid. For patients taking other form of ASMs we typically support the use of 2 mg/day of folic acid, until more literature is available on the least amount of folic acid that can enhance cognition.

## Discussion

Pregnancy registries have largely contributed to ASM management in WWE through the evidence of MCM risks. This has been further expanded by the growing evidence of cognitive, behavioral, and emotional effects of *in utero* ASM exposure provided by studies such as the NEAD study and the Norwegian Mother and Child Cohort Study.

Prescribing practices documented in the North American, EURAP, and Australian Registries have shown drastic changes over the last 5–10 years, with lamotrigine and levetiracetam now being the most prescribed ASMs. Recent data from the EURAP registry has shown that in fact these new practices have led to a statically significant reduction in MCM worldwide (7). With this change in practice, other impacts need to be considered and discussed with patients regarding children exposed to ASMs *in utero* such as lamotrigine, even though they may have a relatively low MCM rates (i.e., abnormal emotional and behavioral development) (11, 18).

It is important to note that there are some patients, particularly those with generalized forms of epilepsy such as Juvenile Myoclonic Epilepsy or Absence Epilepsy, in whom ASMs such as lamotrigine and levetiracetam may not be as effective in controlling seizures as VPA (35, 36). If the patient's seizures are not controlled by less teratogenic ASMs and VPA needs to be used, it is important to find the lowest effective dosage of this ASM to reduce the chances of MCMs as well as cognitive and behavioral deficits.

Even if a woman expresses no desire to become pregnant, all efforts should be made to change the ASM to one with less teratogenic potential to account for unintended pregnancies. It is also recommended that WWE in the reproductive age group take folic acid on a daily basis, particularly if they are sexually active, as this vitamin has been shown to reduce neural tube defects in the general population and enhance cognition in children exposed to ASMs *in utero*. Further research is needed to better understand the dosages of folic acid that provide the maximal benefit. In addition, there are a growing number of ASMs which were introduced to the market after the year 2000 that have unknown teratogenic and cognitive affects. These newer ASMs should be used with caution for WWE until more information is available.

Broadly, epilepsy management is complicated without even considering the sex differences between males and females. In treating WWE, the goal is to reduce the chances of MCM and enhance cognitive development in the fetus who is exposed to ASMs (1, 23).

## Author Contributions

RS: article conception and writing of the manuscript. HM: writing of the manuscript.

## Conflict of Interest

The authors declare that the research was conducted in the absence of any commercial or financial relationships that could be construed as a potential conflict of interest.
